# Mass Production of Early-Stage Bone-Marrow-Derived Mesenchymal Stem Cells of Rat Using Gelatin-Coated Matrix

**DOI:** 10.1155/2013/347618

**Published:** 2013-10-31

**Authors:** Young Hyun Park, Jung Im Yun, Na Rae Han, Hye Jin Park, Ji Yeon Ahn, Choonghyo Kim, Jung Hoon Choi, Eunsong Lee, Jeong Mook Lim, Seung Tae Lee

**Affiliations:** ^1^Stem Cell and Bioevaluation, WCU Biomodulation Program, Seoul National University, Seoul 151-921, Republic of Korea; ^2^Department of Agricultural Biotechnology, College of Agricultural Life and Science, Seoul National University, Seoul 151-921, Republic of Korea; ^3^Department of Animal Biotechnology, College of Animal Life Science, Kangwon National University, Chuncheon 200-701, Republic of Korea; ^4^Cancer Research Institute, College of Medicine, Seoul National University, Seoul 110-799, Republic of Korea; ^5^Department of Neurosurgery, Kangwon National University Hospital, School of Medicine, Kangwon National University, Chuncheon 200-701, Republic of Korea; ^6^College of Veterinary Medicine and Institute of Veterinary Science, Kangwon National University, Chuncheon 200-701, Republic of Korea

## Abstract

Although preparation of early-stage bone-marrow-derived mesenchymal stem cells (BM-MSCs) is critical for successful cell transplantation therapy, no culture system offers a sufficient number of early-stage BM-MSCs for cell transplantation. Accordingly, we developed a culture system capable of producing a large number of early-stage BM-MSCs by using gelatin-coated matrix. The greatest retrieval and proliferation rates of the earliest-stage rat BM-MSCs were detected in bone-marrow-derived cells cultured on 1% (wt/v) gelatin-coated matrix, which showed significantly greater colony forming unit-fibroblast number, diameter, and total cell number. Moreover, continuous culture of the earliest-stage BM-MSCs on 1% (wt/v) gelatin-coated matrix resulted in a maximum of 21.2 ± 2.7 fold increase in the cumulative total number of early-stage BM-MSCs at passage 5. BM-MSCs generated in large quantities due to a reduced doubling time and an increased yield of cell population in S/G2/M phase showed typical fibroblast-like morphology and no significant differences in BM-MSC-related surface marker expression and differentiation potential, except for an increased ratio of differentiation into a neurogenic lineage. The use of gelatin-coated matrix in the retrieval and culture of BM-MSCs contributes greatly to the effective isolation and mass production of early-stage BM-MSCs.

## 1. Introduction 

Mesenchymal stem cells (MSCs) are fibroblast-like cells with the potential to differentiate into multilineage precursor cells [1, 2] and to develop immunomodulatory functions [3, 4]. Among the various sources of MSCs, bone marrow is an especially rich and high-quality source in adults [5]. Therefore, bone-marrow-derived mesenchymal stem cells (BM-MSCs) are used as a therapeutic tool in regenerative medicine [6]. Generally, mass production of early-stage BM-MSCs [7, 8] has been regarded as a key factor for successful cell therapy [9]. However, despite many trials, it has not been possible to obtain a sufficient number of BM-MSCs without long-term *in vitro* culture extinguishing their potentials. Accordingly, we aimed to develop a method for efficiently producing a large number of BM-MSCs in early passages through extracellular matrix derived from gelatin. We identified positive effects of gelatin-coated matrix on the proliferation of primary BM-MSCs without loss of differentiation potential into lineage cells.

## 2. Methods

### 2.1. Animals

Three-week-old male Sprague-Dawley (SD) rats (Japan SLC Inc., Hamamatsu, Japan) were used as bone marrow cell donors. The Animal Care and Use Guidelines of Kangwon National University were adjusted to accommodate all animal housing, handling, and experimental procedures, which were approved by the Institutional Animal Care and Use Committee (IACUC) of Kangwon National University (IACUC approval no. KW-121101-1).

### 2.2. Harvest of Bone-Marrow-Derived Primary Cells

SD rats were sacrificed by CO_2_ asphyxiation; femur and tibias were dissected from both legs and washed with 1% (v/v) Dulbecco's phosphate-buffered saline (DPBS; Welgene Inc., Daegu, Korea) containing antibiotic-antimycotic (Gibco Invitrogen, Grand Island, NY, USA). Muscle on the bones was removed as cleanly as possible. The marrow cavity was exposed by cutting the spongious end of each bone, and the bone marrow-derived primary cells were retrieved by flushing each bone with 2% (v/v) heat-inactivated fetal bovine serum (FBS; HyClone, Logan, UT, USA) containing DPBS. The red blood cells (RBCs) in the collected bone-marrow-derived primary cells were removed using RBC lysis buffer (Sigma-Aldrich, St. Louis, MO, USA). The RBC-free bone-marrow-derived primary cells were counted using a hemocytometer and adjusted for use in subsequent experiments.

### 2.3. Preparation of Gelatin-Coated Culture Dish

Bovine skin-derived gelatin powder (Sigma-Aldrich) was dissolved in deionized water at 100°C, and prepared gelatin solution was stored at 4°C. Subsequently, culture dish was coated with prewarmed gelatin solution for 10 minutes at room temperature, and remaining gelatin solution in culture dish was removed without rinsing. After drying, gelatin-coated culture dish was immediately adjusted to following experiments.

### 2.4. Isolation and Culture of BM-MSC

RBC-free bone marrow-derived primary cells (5 × 10^6^) were cultured on 0, 0.1, 0.5, 1, and 2% (wt/v) gelatin-coated 100 mm tissue culture dishes in low glucose Dulbecco's modified Eagle's Medium (LG-DMEM; Welgene Inc.) supplemented with 10% (v/v) heat-inactivated FBS and 1% (v/v) antibiotic-antimycotic at 37°C under 5% CO_2_ in a humidified chamber. After 2 days, nonadherent cells were removed, and medium changes were performed at 2-3-day intervals. At 14 days of culture, confluent cells were dissociated by 0.25% trypsin-EDTA (Gibco Invitrogen), and cells were enumerated using a hemocytometer. Subsequently, 2 × 10^5^ BM-MSCs cultured under each gelatin-coating condition were reseeded continuously and cultured under the same conditions by the fifth passage. 

### 2.5. Crystal Violet Staining and Colony Forming Unit-Fibroblast (CFU-F) Assay

Harvested RBC-free bone-marrow-derived primary cells were cultured for 7 days on 60 mm culture plates coated with various gelatin concentrations in culture medium consisting of LG-DMEM containing 10% (v/v) heat-inactivated FBS and 1% (v/v) antibiotic-antimycotic. At 7 days of culture, cells washed with DPBS were fixed with 4% (v/v) paraformaldehyde (Junsei Chemical Co., Ltd., Chuo-ku, Japan) for 15 minutes at room temperature. Subsequently, fixed cells were stained by incubating for 5 minutes at room temperature in 0.5% (wt/v) crystal violet (Sigma-Aldrich) solution. Cells stained positively were washed twice with distilled water. A CFU-F was defined as a group of at least 16 cells within a circular area [10]. Subsequently, the numbers and sizes of CFU-F were analyzed using the Image and Microscope Technology (IMT) solution software (IMT i-solution Inc., Vancouver, Canada) under an inverted microscope (CKX-41; Olympus, Tokyo, Japan). 

### 2.6. Calculation of Doubling Time

BM-MSCs were seeded on 100 mm culture plates at a density of 2 × 10^5^ cells. When BM-MSCs were 80% confluent, they were trypsinized, counted, and reseeded at the same density in culture plates. The doubling time of BM-MSCs per passage was calculated by *t*log⁡_2_/(log⁡⁡*N*_*t*_ − log⁡⁡*N*_0_), where *t* is time to confluence, *N*_*t*_ is the number of cells at the end of growth period, and *N*_0_ is the initial number of cells. 

### 2.7. Flow Cytometry Analysis

Undifferentiated or differentiated BM-MSCs were fixed in a 4% (v/v) paraformaldehyde fixative solution. Undifferentiated BM-MSCs were washed in ice cold DPBS and stained for 45 minutes at 4°C in 2% (v/v) FBS-containing DPBS-diluted APC-conjugated anti-CD90 (mesenchymal stem cell-specific marker; BioLegend, San Diego, CA), APC-conjugated anti-CD29 (mesenchymal stem cell-specific marker; BioLegend), FITC-conjugated anti-CD45 (hematopoietic cell-specific marker; BioLegend), and FITC-conjugated anti-CD31 (endothelial cell-specific marker; Abcam, Cambridge, UK) antibodies, respectively. Moreover, to identify the differentiation of BM-MSCs into neuronal lineage cells, APC-conjugated anti-Nestin (BD Biosciences, San Jose, CA) antibody diluted in DPBS supplemented with 0.01% (v/v) Triton-X 100 (Sigma-Aldrich) was added to differentiated BM-MSCs, followed by incubation for 45 minutes at 4°C. Detailed information and dilutions of the primary antibodies are listed in Supplementary Table 1 available online at http://dx.doi.org/10.1155/2013/347618. Stained undifferentiated or differentiated BM-MSCs washed in DPBS were sorted using a FACSCalibur (Becton, Dickinson and Co., Franklin Lakes, NJ), and data analyses were conducted using the BD CellQuest pro software (Becton, Dickinson and Co.).

### 2.8. Cell Cycle Analysis

BM-MSCs fixed with 70% (v/v) ethanol were stained with 1 mL of propidium iodide (PI; Sigma-Aldrich) staining solution consisting of DPBS supplemented with 0.1% (v/v) Triton X-100 (Sigma-Aldrich), 200 *μ*g/mL RNase (Invitrogen, Carlsbad, CA, USA) and 20 ng/mL PI (Sigma-Aldrich). Subsequently, cell cycle status was analyzed by measuring PI fluorescence intensity of the stained BM-MSCs through flow cytometer.

### 2.9. Real-Time PCR

Total mRNA was extracted from the BM-MSCs cultured with or without gelatin matrix until passage 5 using the Qiagen RNeasy minikit (Qiagen, Valencia, CA, USA), according to the manufacturer's instructions. The prepared mRNA was used to create cDNA using the Superscript III First-Strand Synthesis System (Invitrogen, Carlsbad, CA, USA). Real-time quantitative PCR was performed using an iCycler iQ real-time PCR detection system (Bio-Rad Laboratories, Hercules, CA), and melting curve data were collected for identifying PCR specificity. Moreover, normalization of the specific gene expression was performed by comparison to the GAPDH mRNA level. Relative mRNA level was presented as 2^−ΔΔCt^, where Ct = threshold cycle for target amplification, ΔCt = Ct_target  gene_ (specific genes for each sample) − Ct_internal  reference_ (GAPDH for each sample), and ΔΔCt = ΔCt_sample_ (treatment sample in each experiment) − ΔCt_calibrator_ (control sample in each experiment). Information of cDNA sequences were obtained from GenBank for rat, and sequences of primers shown in Supplementary Table 2 were designed by Primer 3 software (Whitehead Institute/MIT Center for Genome Research).

### 2.10. Adipogenic Differentiation Potential Analysis of BM-MSCs

The differentiation of BM-MSCs into adipocytes was performed by incubating cells for 3 weeks in adipogenic differentiation medium consisting of high glucose DMEM (HG-DMEM; Welgene Inc.) supplemented with 10% (v/v) heat-inactivated FBS, 0.5 mM isobutylmethylxanthine (Sigma-Aldrich), 200 *μ*M indomethacin (Sigma-Aldrich), 1 *μ*M dexamethasone (Sigma-Aldrich), 10 *μ*g/mL human insulin (Sigma-Aldrich), and 1% (v/v) antibiotic-antimycotic; the medium was changed every third day. Fixation of BM-MSCs undergoing adipogenic differentiation was conducted for 1 hour at room temperature in a 10% (v/v) paraformaldehyde fixative solution. The fixed cells were washed with 60% (v/v) isopropyl alcohol (Sigma-Aldrich), and the washed cells were completely dried. The differentiated BM-MSCs were stained for 10 minutes at room temperature in an Oil Red O (Sigma-Aldrich) solution consisting of distilled water containing 60% (v/v) isopropyl alcohol and 0.3% (wt/v) Oil Red O reagent. After rinsing four times with distilled water, Oil Red O reagent in the stained cells was extracted by treatment with isopropyl alcohol for 15 minutes. Then, the extracted solution was transferred to 96-well plates, and absorption at 490 nm was measured using a microplate reader (Epoch; Biotek, Winooski, VT, USA). 

### 2.11. The Osteogenic Differentiation Potential of BM-MSCs

Incubation of BM-MSCs for 2 weeks in osteogenic differentiation medium consisting of LG-DMEM supplemented with 10% (v/v) heat-inactivated FBS, 100 nm dexamethasone, 10 mM *β*-glycerolphosphate (Sigma-Aldrich), 50 *μ*M ascorbate-2-phosphate (Sigma-Aldrich), and 1% (v/v) antibiotic-antimycotic was conducted to induce differentiation into osteoblasts. Then, BM-MSCs experiencing osteogenic differentiation were washed with DPBS, fixed with cold 70% (v/v) ethanol, and rinsed with excess distilled water. Alizarin red staining (ARS) of differentiated BM-MSCs was conducted for 10 minutes in a 2% (wt/v) ARS solution (Sigma-Aldrich), and stained cells were washed with distilled water four times to remove unincorporated alizarin Red dye. After drying completely, the deposited alizarin-calcium complexes were extracted with 2 mM NaHPO_4_ (Sigma-Aldrich) solution containing 10% (wt/v) cetyl-pyridinium chloride (CPC; Sigma-Aldrich). Subsequently, the extracted solution was transferred into 96-well plates, and quantification of Alizarin Red dye was conducted by measuring absorption at 550 nm using a microplate reader (Biotek). 

### 2.12. Chondrogenic Differentiation of BM-MSCs

Differentiation of BM-MSCs into chondrocytes was conducted using a StemPro Chondrogenesis Differentiation Kit (Gibco Invitrogen), according to the manufacturer's instructions. After 3 weeks of differentiation, differentiated cell pellets were fixed with 4% (v/v) paraformaldehyde and embedded in paraffin. Differentiation into chondrocytes was identified by alcian blue staining (Sigma-Aldrich) under a microscope (BX-43; Olympus). 

### 2.13. Analysis of Neurogenic Differentiation Potential of BM-MSCs

BM-MSCs were exposed for 5 h to preinduction medium consisting of HG-DMEM containing 0.1 mM 2-mercaptoethanol (Gibco Invitrogen) and 2% (v/v) dimethylsulfoxide (DMSO; Sigma-Aldrich). Then, they were maintained for 7 days in neuronal induction medium consisting of HG-DMEM supplemented with 10% (v/v) heat-inactivated FBS, 10 *μ*g/L basic fibroblast growth factor (b-FGF; R&D Systems, Inc., Minneapolis, MN), 10 *μ*g/L human epidermal growth factor (hEGF; R&D Systems, Inc.), 1 mM dibutyryl cyclic AMP (dbcAMP; Sigma-Aldrich), and 0.5 mM isobutylmethylxanthine (IBMX; Sigma-Aldrich). BM-MSCs undergoing neurogenic differentiation were washed with DPBS (Welgene Inc.) and were dissociated by 0.25% trypsin-EDTA. Subsequently, the dissociated cells were subjected to flow cytometry analysis.

### 2.14. Statistical Analysis

The Statistical Analysis System (SAS) program was used for analysis of all numerical data. Each treatment was compared using the least-square difference or Duncan's method, and analysis of variance (ANOVA) in the SAS package was used to determine the significance of the main effects. The level of significant differences was set at *P* < 0.05.

## 3. Results

### 3.1. Effects of Gelatin-Coated Matrix on Retrieval and Proliferation of the Earliest-Stage MSCs from Bone-Marrow-Derived Primary Cells

**Figure 1:**
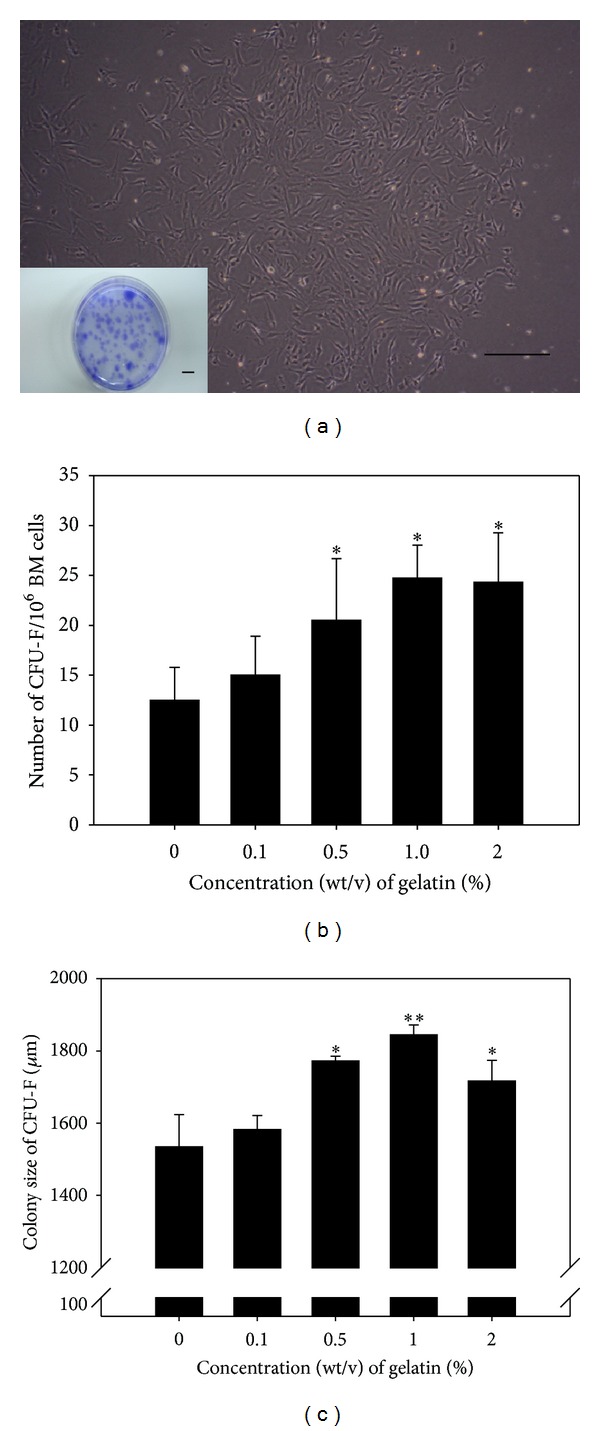
The effect of the gelatin concentration of gelatin-coated two-dimensional (2D) matrix culture dishes on the number and colony size of colony forming unit-fibroblasts (CFU-Fs) derived from mesenchymal stem cells obtained from rat bone marrow primary cell populations. Bone-marrow-derived primary cells were seeded onto culture plates coated with 0, 0.1, 0.5, 1 and 2% (wt/v) gelatin and cultured for 7 days. The CFU-Fs were analyzed using the Image and Microscope Technology (IMT) solution software under an inverted microscope. (a) The CFU-Fs had a typical fibroblastic morphology and were stained by crystal violet. (b) A significant increase in the number of CFU-Fs was detected in bone-marrow-derived mesenchymal stem cells (BM-MSCs) cultured on 0.5, 1 and 2% (wt/v) gelatin matrices. (c) The CFU-Fs with the largest diameters were those of BM-MSCs cultured on 1% (wt/v) gelatin matrix. All data shown are means ± S.D. of four independent experiments. Scale bar (large), 400 *μ*m. Scale bar (small), 1 cm. ^∗,∗∗^ *P* < 0.05.

**Figure 2:**
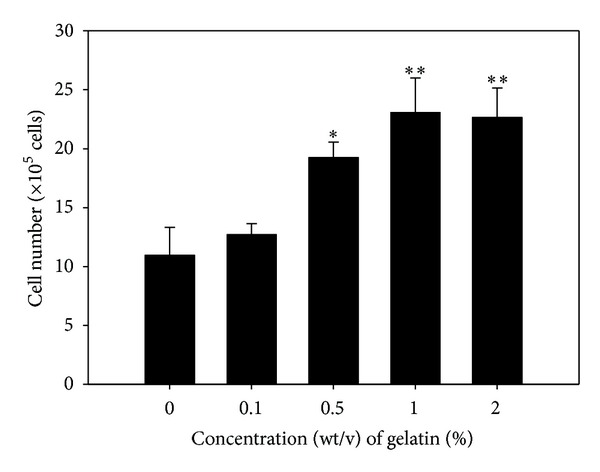
Quantification of BM-MSCs cultured for 14 d on 2D matrices coated with various gelatin concentrations. Bone-marrow-derived primary cells were cultured on culture plates coated with 0, 0.1, 0.5, 1 and 2% (wt/v) gelatin and the cells stained negatively with trypan blue were enumerated using a hemocytometer. The highest numbers of BM-MSCs were obtained from bone-marrow-derived primary cells cultured on 1 and 2% (wt/v) gelatin-coated culture dishes. All data shown are means ± S.D. of five independent experiments. ^∗,∗∗^ *P* < 0.05.

To determine whether gelatin stimulates proliferation of only MSCs among the diverse types of bone-marrow-derived primary cells, bone-marrow-derived primary cells were cultured on 0, 0.1, 0.5, 1, and 2% (wt/v) gelatin-coated matrix for 7 days, and the numbers and diameters of MSC-derived CFU-F in cell groups within a circular area (Figure 1(a)) were measured. A significant increase in the number of CFU-Fs was detected in bone-marrow-derived primary cells cultured on 0.5, 1, and 2% (wt/v) gelatin-coated matrix (Figure 1(b)). Furthermore, significantly larger colonies of CFU-Fs were observed for bone-marrow-derived primary cells cultured on 1% (wt/v) gelatin-coated matrix (Figure 1(c)). At each gelatin concentration, the CFU-Fs maintained culturability on the gelatin-coated matrix for 7 days. On the 1% (wt/v) gelatin-coated matrix, the total number of MSCs among the bone-marrow-derived cells was significantly increased by 2.16 ± 0.44 fold compared to cells grown in the absence of gelatin-coating (Figure 2). These results demonstrated that a matrix coated with 1% (wt/v) gelatin could greatly increase the yield of the earliest-stage MSCs by improving retrieval and proliferation of MSCs included in bone-marrow-derived primary cells.

### 3.2. Effects of Gelatin-Coated Matrix on Production of Early-Stage BM-MSCs

**Figure 3:**
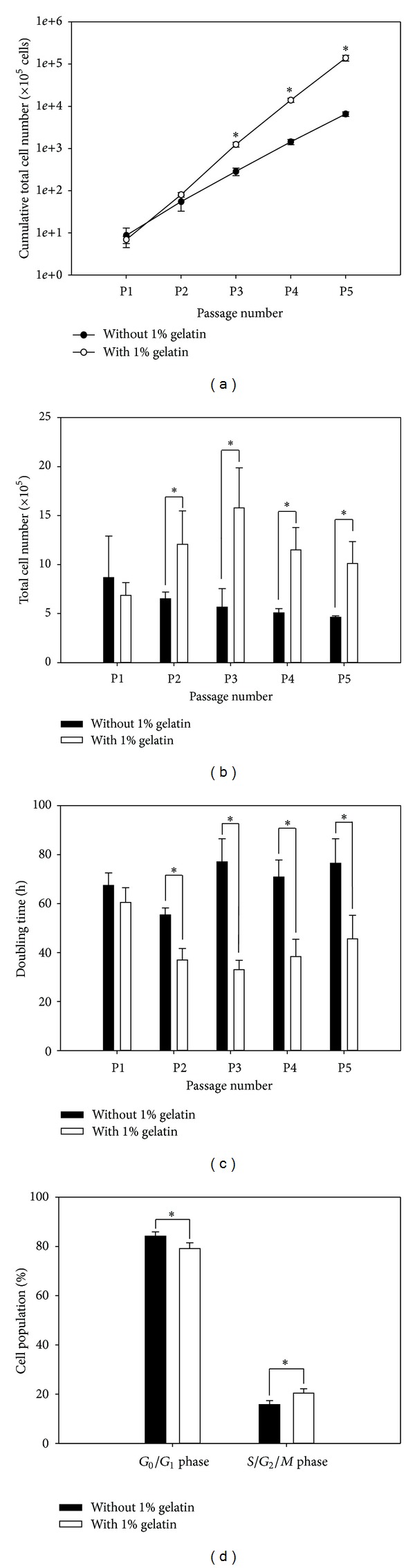
The effect of gelatin-coated matrix on the proliferation of BM-MSCs. BM-MSCs were from bone-marrow-derived primary cell populations cultured on dishes coated without or with 1% (wt/v) gelatin for 14 days. Subsequently, 2 × 10^5^ BM-MSCs derived from each 2D matrix condition per passage were cultured continuously on 100 mm culture dishes coated without or with 1% (wt/v) gelatin by passage 5. A hemocytometer was used to count the number of negatively trypan blue-stained BM-MSCs obtained at each passage, and the doubling time of BM-MSCs per passage was calculated by *t*log⁡_2_/(log⁡⁡*N*_*t*_ − log⁡⁡*N*_0_), where *t* is time to confluence, *N*_*t*_ is the final cell number, and *N*_0_ is the initial cell number. Cell cycle status was investigated in BM-MSCs at passage 5 through FACS analysis. As the results, gelatin-mediated significant increases in cumulative total cell number (a) and total cell number (b) were detected after passages 3 and 2, respectively. A significant decrease in doubling time was found after passage 2 in BM-MSC cultures on a 1% (wt/v) gelatin-coated 2D matrix, compared to cells cultured without gelatin (c). In case of cell cycle status, culture of BM-MSCs on a 1% (wt/v) gelatin-coated 2D matrix increased significantly the yield of BM-MSCs in S/G2/M phase and decreased significantly the yield of BM-MSCs in G0/G1 phase, compared to cell cultured without gelatin (d). All data shown are means ± S.D. of five ((a), (b), and (c)) or three (d) independent experiments. *P < 0.05.

In general, a sufficient number of cells is required for cell transplantation into target tissue [11–13]. In this study, 2 × 10^5^ earliest-stage BM-MSCs retrieved and proliferated in the presence or absence of 1% (wt/v) gelatin-coated matrix were cultured in conditions with or without gelatin during early passages (up to passage 5), and the cumulative total number (per passage), total number (per passage), doubling time (per passage), and cell cycle status (at passage 5) of cultured BM-MSCs were evaluated. A significant increase in cumulative total cell number was detected after passage 3 in BM-MSCs cultured in the presence of 1% (wt/v) gelatin, compared to BM-MSCs cultured in the absence of gelatin (Figure 3(a)); at passage 5, the cumulative total number of BM-MSCs cultured with gelatin was a maximum of 21.2 ± 2.7 times greater than BM-MSCs cultured without gelatin. This result was supported by the significantly higher total number (Figure 3(b)) and lower doubling time (Figure 3(c)) after passage 2 and higher yield of cell population in S/G2/M phase (Figure 3(d)) in BM-MSCs cultured in the presence of gelatin compared to those cultured in its absence. These results demonstrated the development of a culture system that supports mass production of early-stage BM-MSCs using a 1% (wt/v) gelatin-coated matrix.

### 3.3. Cellular Morphology, Surface Marker Expression, and Differentiation Potential of Early-Stage BM-MSCs Cultured in a Gelatin-Coated Matrix

To determine whether cellular characteristics were altered in BM-MSCs maintained in the presence of gelatin-coated matrix, we investigated the cellular morphology, surface marker expression, and differentiation potential of BM-MSCs proliferated on 1% (wt/v) gelatin-coated cultures at passage 5. 

**Figure 4:**
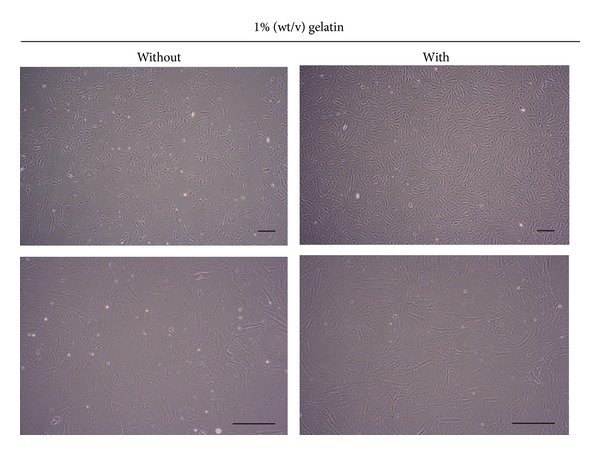
The morphology of BM-MSCs cultured on a 2D matrix coated with 1% (wt/v) gelatin by passage 5. Compared to BM-MSCs cultured on dishes without a gelatin coating, a typical fibroblast-like morphology was observed in BM-MSCs cultured on 1% (wt/v) gelatin-coated culture dishes. Magnifications of photographs in top and bottom panel are ×40 and ×100, respectively. Scale bar, 200 *μ*m.

**Figure 5:**
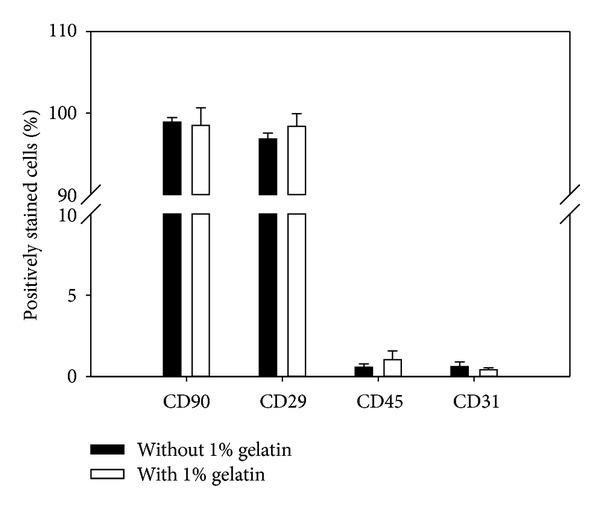
Expression of mesenchymal stem cell-related surface marker proteins in BM-MSCs on culture dishes coated without or with 1% (wt/v) gelatin by passage 5. Cultured BM-MSCs were stained with fluorescence-conjugated primary antibodies against CD90 and CD29 (mesenchymal stem cell-specific markers), CD45 (hematopoietic stem cell-specific marker), and CD31 (endothelial cell-specific marker) and analyzed using flow cytometry. No significant differences in the expression of BM-MSC-specific, hematopoietic stem cell-specific, and endothelial cell-specific surface marker proteins were induced by gelatin. All data shown are means ± S.D. of three independent experiments.

Typical fibroblast-like morphology of BM-MSCs was observed both in BM-MSCs proliferated in large quantity on gelatin-coated matrix and in BM-MSCs cultured in the absence of gelatin (Figure 4). Moreover, surface marker expression analysis (Supplementary Figure 1 and Figure 5) revealed that BM-MSCs proliferated in large quantities under gelatin-coated matrix conditions expressed the MSC-specific marker genes CD44 and CD105 and were positive for the MSC-specific marker proteins CD90 (98.48 ± 2.15%) and CD29 (98.36 ± 1.57%), but not for a hematopoietic lineage marker protein (CD45; 1.03 ± 0.54%) or an endothelial lineage marker protein (CD31; 0.42 ± 0.12%). Moreover, no significant increase or decrease in total surface marker gene and protein expression was detected in BM-MSCs proliferated in large quantities under gelatin-coated matrix conditions, compared to BM-MSCs cultured under non-gelatin-coated matrix conditions.

**Figure 6:**
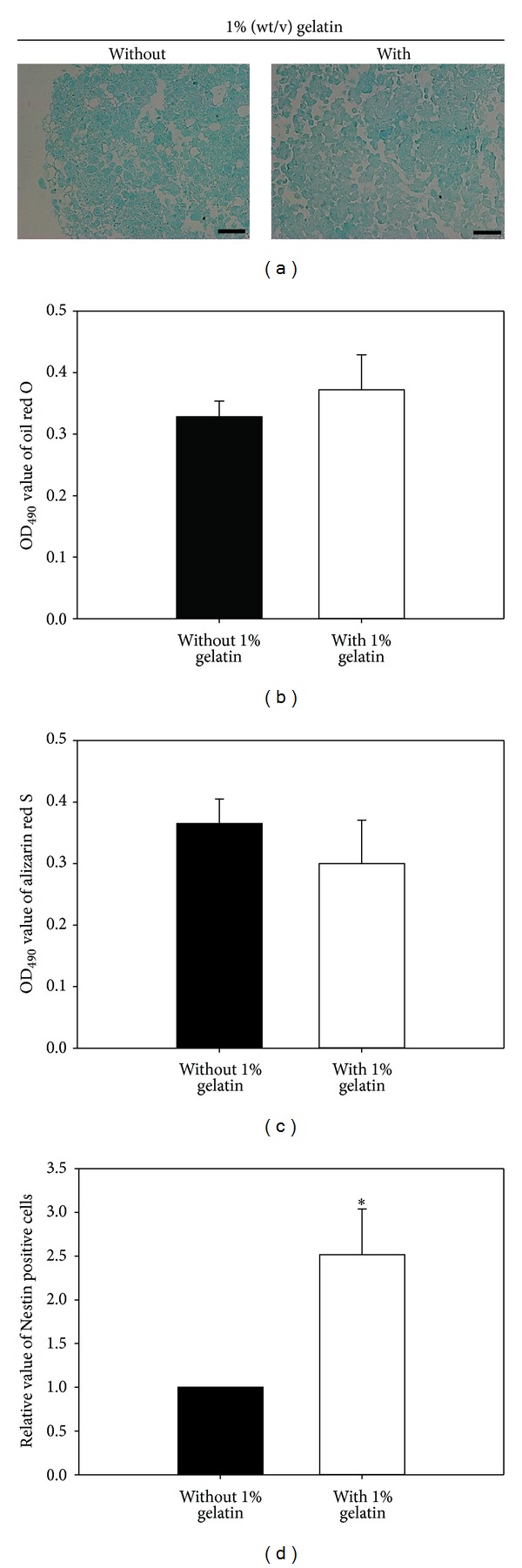
Effects of gelatin-coated matrix on the differentiation potential of BM-MSCs. By passage 5, BM-MSCs were cultured on dishes coated without or with 1% (wt/v) gelatin. (a) For analysis of chondrogenic differentiation potential, BM-MSCs were cultured on a 1% (wt/v) gelatin matrix using a chondrogenesis differentiation kit for 3 weeks, and differentiation into chondrocytes was assessed by alcian blue staining. (b) For analysis of adipogenic differentiation potential, BM-MSCs were incubated for 3 weeks in adipogenic differentiation medium. BM-MSCs that differentiated into adipocytes were identified by Oil red O staining and quantified by measuring the absorbance at 490 nm. There was no significant difference in adipogenic differentiation potential between BM-MSCs cultured without and with 1% (wt/v) gelatin. (c) For analysis of osteogenic differentiation potential, BM-MSCs were incubated in osteogenic differentiation medium for 2 weeks. BM-MSCs that differentiated into osteoblasts were identified by alizarin red staining (ARS), which stains calcium deposits in the differentiated cells, and quantified by measuring the absorbance at 550 nm. There was no significant difference in osteogenic differentiation potential between BM-MSCs cultured with and without 1% (wt/v) gelatin. (d) For analysis of neurogenic differentiation potential, BM-MSCs were cultured for 7 days in neurogenic differentiation medium. BM-MSCs that differentiated into neurogenic cells were stained with a fluorescence-conjugated primary antibody against Nestin (neural lineage marker) and assessed by flow cytometry. A significantly higher percentage of Nestin-positive cells was detected in BM-MSCs cultured on 1% (wt/v) gelatin matrix than in those cultured on plates without gelatin. All data shown are means ± S.D. of three (d) or four ((b) and (c)) independent experiments. *P < 0.05. Scale bar, 50 *μ*m.

 We assessed whether BM-MSCs proliferated in large quantities on gelatin-coated matrix could differentiate into chondrogenic lineages by alcian blue staining (Figure 6(a)). We found that signaling derived from gelatin-coated matrix did not induce any significant difference in the ratio of BM-MSC differentiation into adipogenic (Figure 6(b)) and osteogenic lineages (Figure 6(c)). In contrast, a significant increase in the differentiation of BM-MSCs into a neurogenic lineage was induced by gelatin (Figure 6(d)). These results demonstrate that *in vitro* culture of BM-MSCs with a gelatin-coated matrix is effective for mass production of early stage BM-MSCs without loss of the original cellular characteristics.

## 4. Discussion

To obtain a large amount of homogeneous MSCs from a variety of sources is very important for successful cell therapy [14]. In particular, in order to harvest effectively MSCs from bone marrow, plastic adherence [10, 15], density gradient centrifugation [16], and immunomagnetic selection methods [17] have been developed. However, these methods have proven to be inefficient due to a low MSC retrieval rate and contamination of the culture by hematopoietic cells [18, 19]. Therefore, development of a reliable and easy method of isolating and expanding a homogenous population of MSCs from bone marrow is critical.

In this study, we designed a cell culture plate coated with a 2D matrix that provides extracellular signaling derived from gelatin, a substance known to improve cell attachment and proliferation of bone marrow-derived MSCs (BM-MSCs). Culturing BM-MSCs on gelatin matrix plates led to twofold higher retrieval of BM-MSCs and a 21-fold increase in BM-MSC production during the early passage period (by passage 5). In other words, a 42-fold increased yield of early-stage BM-MSCs could be produced from bone marrow using the gelatin matrix. Moreover, gelatin did not affect the cellular characteristics and differentiation potential of BM-MSCs.

In this study, we observed an increased number and diameter of MSC-derived CFU-Fs and an increased total number of BM-MSCs cultured on gelatin matrix. These results demonstrate the positive effects of gelatin on the purification of MSCs from bone-marrow-derived primary cell populations and proliferation of BM-MSCs in culture. Although similar effects of gelatin have been reported in other cell types [20], the BM-MSC yields obtained were not reported. A gelatin-mediated decrease in BM-MSC doubling time (Figure 3(c)) and increase in the percentage of S/G2/M phase BM-MSCs (Figure 3(d)) may explain the increase in BM-MSC proliferation, which resulted in mass production of early stage BM-MSCs. Further investigation is required to elucidate the gelatin-mediated regulation of intracytoplasmic events at the molecular level. In addition, mass production of early stage BM-MSCs may be resulted from synergistic effects of MSC proliferation (Figure 3) and adhesion (data not shown) promoted by gelatin-coated matrix. 

Due to the high adherence rate of cells [21–23] to gelatin during MSC purification from the bone-marrow-derived primary cell population, it is possible that some adherent nonmesenchymal cells were counted falsely as CFU-Fs or increased the size of CFU-Fs, thereby affecting the results [24]. Accordingly, it should be noted that contamination of MSC cultures with nonmesenchymal cells may influence their growth kinetics and increase the variability of growth [25, 26]. However, compared to cells cultured on a nongelatin matrix, our flow cytometry analysis showed no significant difference in MSC-related surface marker expression on BM-MSCs cultured on gelatin matrix at passage 0 (data not shown) and passage 5 (Figure 5). Therefore, gelatin does not increase the heterogeneity or decrease the homogeneity of MSC populations produced from bone-marrow-derived primary cell populations. 

Generally, biophysical and biochemical factors consisting of stem cell niche have been regarded as important regulators in the decision of cell fate. In the previous reports, osteogenic or neuronal differentiations of pluripotent stem cells [27–29] or multipotent adult stem cells [30–33] were promoted by bone-like hard or brain-like soft stiffness, and these facts demonstrate that substrate stiffness make it a key role in guiding differentiation of pluripotent stem cells or multipotent adult stem cells into specific lineage cells. In this study, Figure 6(d) showed that gelatin-coated matrix could improve the neurogenic differentiation potential in MSCs with multipotency, indicating that gelatin substrate may have brain-like soft stiffness. In addition, gelatin may contain unidentified motif that can stimulate signal pathway related with neuronal differentiation, and additional studies about these should be requested further.

Previous studies have shown that ECM proteins (such as collagen type I and IV, laminin, and fibronectin) stimulate neural stem/progenitor cell differentiation into neuronal lineage cells [34–36]. Likewise, our data suggest that gelatin-coated matrix can significantly improve the differentiation of BM-MSCs into neuronal lineage cells; however, no effects of gelatin were observed in terms of the adipogenic, osteogenic, and chondrogenic differentiation potential of BM-MSCs. Therefore, this favorable neurogenic microenvironment may in future be a useful tool for the neuronal differentiation of BM-MSCs for the treatment of neuronal disease.

## 5. Conclusion

The ECM is involved in cell growth, movement, differentiation, and undifferentiation [29, 37–39]. Gelatin, a hydrolyzed form of collagen IV, is a component of the ECM that can be used readily and at low cost. Using a 1% (wt/v) gelatin-coated matrix, we established a simple culture system for production of large quantities of early-stage BM-MSCs. Effective purification and proliferation of MSCs from bone-marrow-derived primary cells will contribute greatly to the development of cell therapy and transplantation using BM-MSCs. 

## Supplementary Material

The Supplementary Materials contain one figure and two tables. In details, Supplementary Figure 1 show a relative expression level of mesenchymal stem cell-specific genes (CD44 and CD105) in BM-MSCs on culture dishes coated without or with 1% (wt/v) gelatin by passage 5, Supplementary Table 1 contain detailed information of primary antibodies used in FACS analysis, and Supplementary Table 2 contain detailed information of oligonucleotide primers and PCR cycling conditions for real-time PCR.
