# Supplementary figures and images for: Mass Production of Early-Stage Bone-Marrow-Derived Mesenchymal Stem Cells of Rat Using Gelatin-Coated Matrix
Source: Biomed Res Int. 2013 Oct 31;2013:347618. doi: 10.1155/2013/347618 (PMC3833006; doi:10.1155/2013/347618)

**(A)**

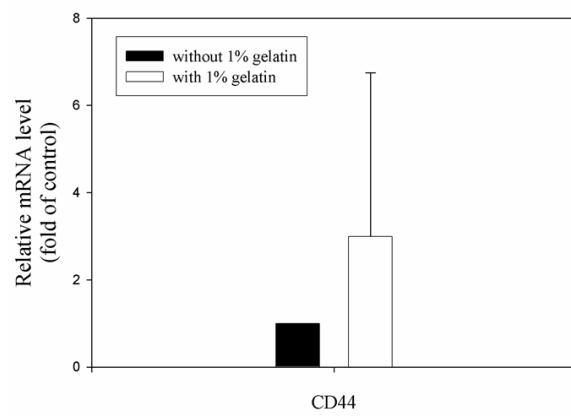

**(B)**

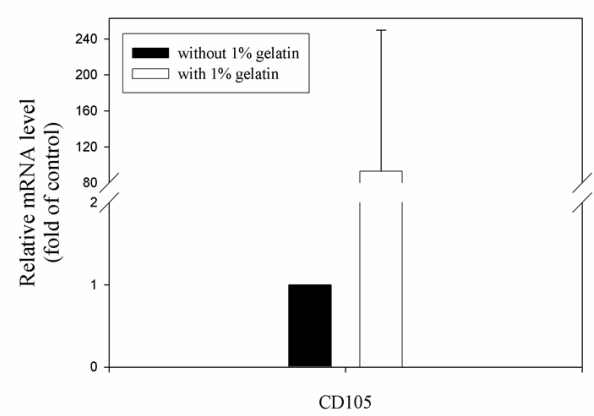

Supplement: Supplementary file 1 — The Supplementary Materials contain one figure and two tables. In details, Supplementary Figure 1 show a relative expression level of mesenchymal stem cell-specific genes (CD44 and CD105) in BM-MSCs on culture dishes coated without or with 1% (wt/v) gelatin by passage 5, Supplementary Table 1 contain detailed information of primary antibodies used in FACS analysis, and Supplementary Table 2 contain detailed information of oligonucleotide primers and PCR cycling conditions for real-time PCR. [file 347618.f1.pdf]
