# Supplementary material for: Mass Production of Early-Stage Bone-Marrow-Derived Mesenchymal Stem Cells of Rat Using Gelatin-Coated Matrix
Source: Biomed Res Int. 2013 Oct 31;2013:347618. doi: 10.1155/2013/347618 (PMC3833006; doi:10.1155/2013/347618)
Supplement: Supplementary file 2 [file 347618.f2.pdf]

Supplementary Table 1. Primary antibodies.

Antibody Name	Clone	Catalog Number	Company	Dilution Rate
APC anti-mous/rat CD29	HMß1-1	102215	Biolegend	1:250
FITC anti-mouse CD31	TLD-3A12	Ab33858	Abcam	1:250
FITC anti-rat CD45	OX-1	202205	Biolegend	1:250
APC anti-rat CD90	OX-7	202526	Biolegend	1:500
Alexa Fluor® 647 anti-rat Nestin	25/NESTIN	560393	BD bioscience	1:20
