# Supplementary material for: Mass Production of Early-Stage Bone-Marrow-Derived Mesenchymal Stem Cells of Rat Using Gelatin-Coated Matrix
Source: Biomed Res Int. 2013 Oct 31;2013:347618. doi: 10.1155/2013/347618 (PMC3833006; doi:10.1155/2013/347618)
Supplement: Supplementary file 3 [file 347618.f3.pdf]

Supplementary Table 2. Oligonucleotide primers and PCR cycling conditions.

Genes	Accession number	Primer sequence		Size (bp)	Temperature (°C)
		Sense (5’>3’)	Anti-sense (5’>3’)		
CD45	NM_012924.2	CAGCCAGTGACAGGTTCCAT	CTGTGCCAGGCTCAACTGTA	159	60
CD105	NM_001010968	GAATGGCAACCACGAACCCA	CCAGGAGACGTACGGGGGAC	195	60
GAPDH	NM_017008.4	GGACCTCATGGCCTACATGG	CCCCTCCTGTTGTTATGGGG	179	60
